# Incidence and Epidemiology of Kidney Infarctions in Germany—A Cohort Study

**DOI:** 10.3390/epidemiologia6020019

**Published:** 2025-04-14

**Authors:** Yannick Rau, Ludwig Matrisch

**Affiliations:** 1 Department of Internal Medicine, Schön Klinik Neustadt, Am Kiebitzberg 10, 23730 Neustadt, Germany; 2 Department of Internal Medicine I, University Medical Center Schleswig-Holstein, Campus Lübeck, Ratzeburger Allee 160, 23538 Lübeck, Germany

**Keywords:** incidence, renal infarctions, kidney infarctions, epidemiology, vascular disease, renal, kidney

## Abstract

Background/Objectives: The aim of this study was to quantify and analyze the incidence and epidemiology of kidney infarctions between 2012 and 2022 in Germany. Methods: We analyzed and extracted data from the national database of federal health reporting. Incidence rates were calculated and stratified by gender and age. Statistical analysis involved linear regression to assess correlations between incidence, age, and reporting year, with significance determined using F-tests and Student’s *t*-tests. Results: From 2012 to 2022, 7983 cases of kidney infarction (4769 male, 3214 female) were identified. The mean incidence was 8.81 per million per year, higher in males (10.7) than females (6.99). Incidence peaked among individuals aged 50–59 years. A significant decrease in incidence over the study period was observed, particularly among males (−2.49 per million per year) compared to females (−0.87 per million per year). Linear regression showed a significant correlation between incidence and age (F(1,6) = 131, *p* < 0.001) and a significant overall incidence decrease over time (F(1,9) = 40.5, *p* < 0.001). Conclusions: This study provides the first nationwide epidemiological data on kidney infarction in a Western country. The downward trend, especially among males, may be due to the improved management of risk factors like atherosclerosis and atrial fibrillation, e.g., through an increase in the prescription of direct anticoagulatory agents. Despite the decrease, kidney infarction remain a significant cause of acute kidney injury. Further research is needed to understand these trends and improve preventive strategies.

## 1. Introduction

Kidney infarction is the result of disruption of renal blood flow with the subsequent lack of oxygenation within the renal tissue. Individual functional units of the kidney, better known as nephrons, are highly susceptible to ischemic states, since, unlike other tissue like muscle fibers, the individual cells neither contain many oxygen reserves nor are they resilient against anaerobic conditions due to a high metabolic turnover. Ischemia therefore quickly results in cell death of renal tissue with the potential consequence of complete kidney failure [1,2].

The etiology of kidney infarction is heterogeneous. One commonly mentioned classification of the possible etiologies is the one proposed by Bourgault et al. They propose four groups: Firstly, kidney infarctions can be caused by a dislocated embolism from the endothelium of the renal arteries [3]. This is most often the result of preexisting atherosclerosis and occurs analogous to, e.g., embolism from carotid or coronary arteries [4]. The second group of kidney infarction causes is the dislocation of an embolus from the heart. Atrial fibrillation contributes to an increased risk of blood clotting within the left atrium. The resulting thrombus may dislocate and become an embolus within the arterial system. This can result in the infarction of different organ systems. Most commonly known are infarctions of the brain, better known as ischemic strokes [5]. However, mesenterial infarctions, infarctions of the spinal cord, or, in the cases of some patients, renal infarctions are also among the results of such embolisms [6,7]. Thirdly, Bourgault et al. mention kidney infarctions due to underlying hypercoagulability disorders causing blood clotting and thereby disrupting blood flow in the renal arteries. Lastly, all other cases are categorized as “apparently idiopathic”. These kidney infarctions may, in some cases, also be iatrogenic. Endovascular interventions can cause embolisms to release and induce infarction [8]. The usage of fibrinolytic agents or anticoagulation are also reported to possibly trigger infarctions via the release of embolisms [4,9].

Patients with a kidney infarction most commonly present with flank pain and preexisting risk factors like atrial fibrillation, hypertension, pre-existing hypercoagulability disorders or obesity [10].

The first reported case of kidney infarction occurred in the year of 1856 by Ludwig Traube in his work about the connection between cardiac and renal diseases [11]. Although diagnostics and preventative measures have changed drastically since then, kidney infarction remains an underestimated disease with possible life changing implications.

The more current literature still describes kidney infarction as a rare disease. A retrospective analysis of cases from Domanovits et al. from 1999 estimates the yearly incidence among emergency department patients at about 0.007%. However, this study was conducted using a monocentric study design, and could only identify 17 patients during a 36-month observation period [12]. The generalizability of the resulting data therefore maybe significantly limited. Another more recent monocentric study performed by Paris et al. in 2006 reported a total of 69 patients with kidney infarctions from 1986 to 2004 within a total patient-population of 18,287 patients. This results in an incidence of approximately 0.3% [8]. Again, the generalizability of such data is impaired by the restrictions of the study’s monocentric design and specific conditions within the analyzed institution.

While most patients with previously healthy kidneys only develop acute kidney injury, especially patients with previously decreased renal function or previously injured kidneys may develop chronic kidney disease and in rare cases even end stage renal failure as a result [2]. The latter is most likely if both kidneys are affected or patients only have one functioning kidney to begin with [13].

Since kidney infarction is still a rare disease, lacking recent studies on its epidemiology on a population-based level and considerations on its impact among today’s population are difficult to make, we decided to close the existing gap in the epidemiological data.

In this study, we use a registry-based approach to quantify the incidence of hospitalized kidney infarctions in Germany from 2012 to 2022. We analyze the potential differences between the sexes and age groups, as well as potential developments over the analyzed period.

## 2. Materials and Methods

### 2.1. Data Collection

Data on the epidemiology and incidence of renal infarctions were retrieved from the Gesundheitsberichterstattung des Bundes (federal health reporting) database of the federal bureau of statistics, Department of the Interior [14]. Every hospital treating patients insured by statutory health insurance in Germany is required to continuously report certain data—including diagnoses and patient characteristics—to state institutions, by law. The reported data are then bundled in the federal health reporting database. We retrieved the data of the International Statistical Classification of Diseases and Related Health Problems (ICD) 10—German modified code as the main treatment diagnosis, representing kidney ischemia and infarction, and prepared it for further analysis. All other patients were excluded. Hospitals are required to provide the diagnosis code upon the patients’ releases. The accumulated incidences therefore actually represent the number of patients with N28.0 as their main treatment diagnosis, released from hospital care within the year of report. Our study therefore does not include patients with occlusion of the renal arteries caused by stenosis due to atherosclerosis itself without embolism, as this would be considered as an ICD-10 German modified Diagnosis I70.1 [15].

For demographic stratification, data were retrieved from the GENESIS database of the federal bureau of statistics of Germany. The GENESIS database is the datahub for state provided statistics in Germany and is maintained by the federal statistics office. It is accessible by anyone through the bureau’s website [16]. To calculate the incidence, we then divided the absolute number of cases by the respective population size for each year, each age group and each sex.

### 2.2. Statistical Analysis

Statistical analysis was performed using Microsoft Excel (Version 2207, Microsoft, Redmond, WA, USA) and jamovi (Version 2.3.18, The Jamovi Project, Sydney, Australia), an open-source tool based on the programming language R, enabling a high degree of reproducibility. Incidence data were transformed into incidence per million per year, using the population data to account for demographic changes. Incidence was then reported, stratified by gender and age. Additionally, linear regression analyses were performed to quantify the correlation between the age groups, years of report, and renal infarction incidence. This was again partly performed for the male and female population alike. Regression model fitness is presented by adjusted Nagelkerke’s R^2^. Furthermore, an F-test was performed on each analysis to further determine the significance of the modeled coefficients.

To compare the two groups’ incidences directly, we also performed Student’s *t*-test. A difference with an alpha error probability of *p* < 0.05 was considered as statistically significant in all analyses.

## 3. Results

During the study period of 2012 to 2022, we identified a total of 7983 (4769 male and 3214 female) patients with kidney infarctions. The largest group of cases was made up by those between the ages of 50 to 59 years old (1533 cases). A total of 1091 of these patients were male. In the female subpopulation, the largest group, with 896 patients, was made up of patients between the ages of 70 to 79 years old.

Stratified for the population size of each year, a mean incidence of 8.81 per one million inhabitants per year could be calculated. An overview of incidences among both sexes and among age groups can be seen in Table 1.

The decrease in incidence among the female population over the observed period accumulates to roughly 0.87 per million per year, while the male population presented a decrease of approximately 2.49 per million per year. The relative decrease also differs respectively, with approximately 21% in the male population and approximately 12% within the female population.

**Figure 1:**
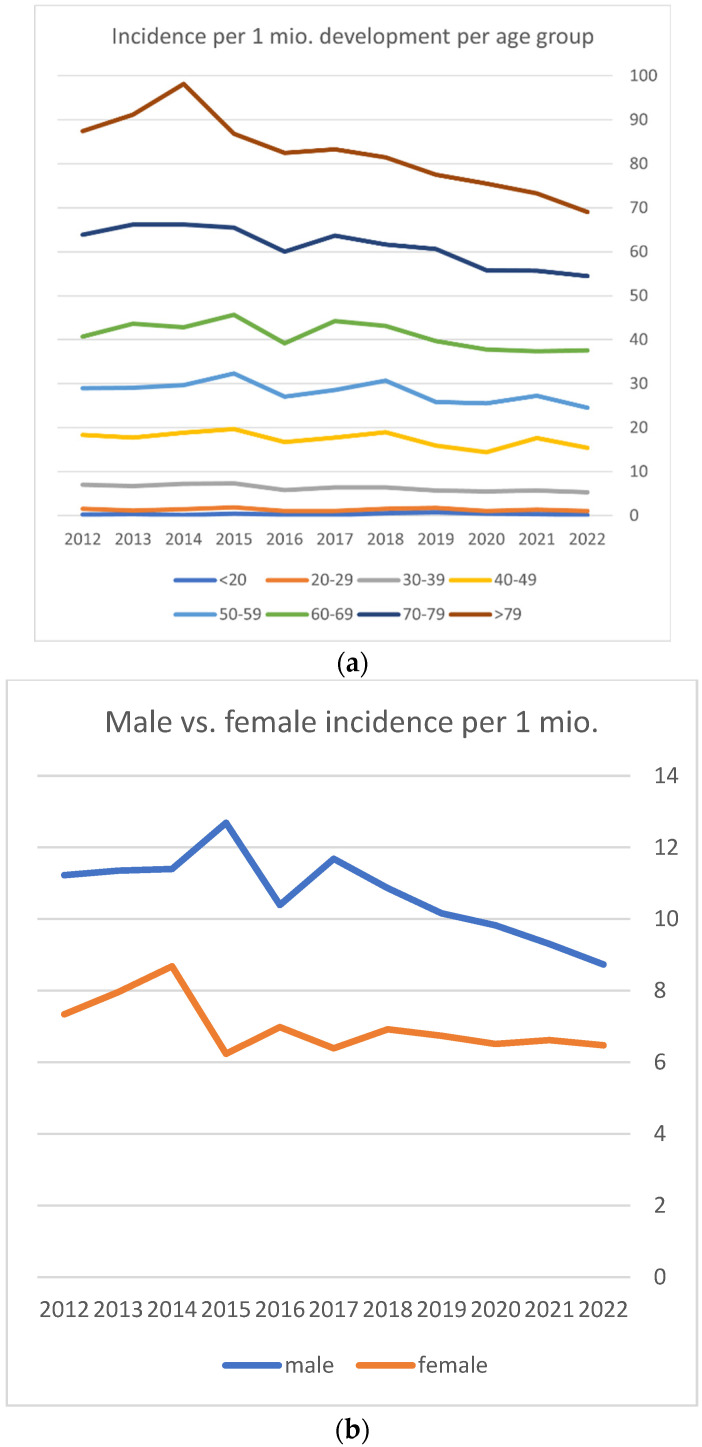
(**a**) Incidence development over time per age group. (**b**) Male and female incidence development over time.

Figure 1a illustrates the incidence development per age group over time. The graphs show that a decrease in incidence over time is particularly prevalent in patients over the age of 80 years old. The absolute and relative decrease in incidence in the other age groups was relatively smaller as can be seen.

When analyzing the relation between age group and incidence via linear regression, a significant correlation can be calculated too with F(1,6) = 131; *p* < 0.001, an adjusted R^2^ of 0.949 and a correlation coefficient of 3.15 per one million per year. The increase in kidney infarction incidence with increasing age can, therefore, also be assumed to be statistically significant.

Figure 1b shows the development of incidence among the male and female population. It shows that the decrease in overall incidence is primarily caused by a decrease in the male population.

When comparing female and male mean incidences via Student’s *t* test, a significant difference between the two means can be assumed (*p* < 0.0001). The same is true when comparing female incidence to the overall incidence (*p* < 0.0001), as well as for male incidence compared to overall incidence (*p* = 0.0002). Distribution was tested prior by the Shapiro–Wilk test (W = 0.98, *p* = 0.917), as well as Levene’s test (F = 2.47, *p* = 0.132). An additional comparison of each incidence per age group divided by sex is shown in Table 2 and shows a significant difference between sexes in the age groups from 30 to 79 years old. A significant correlation between sex and the occurrence of kidney infarction can be assumed.

**Figure 2:**
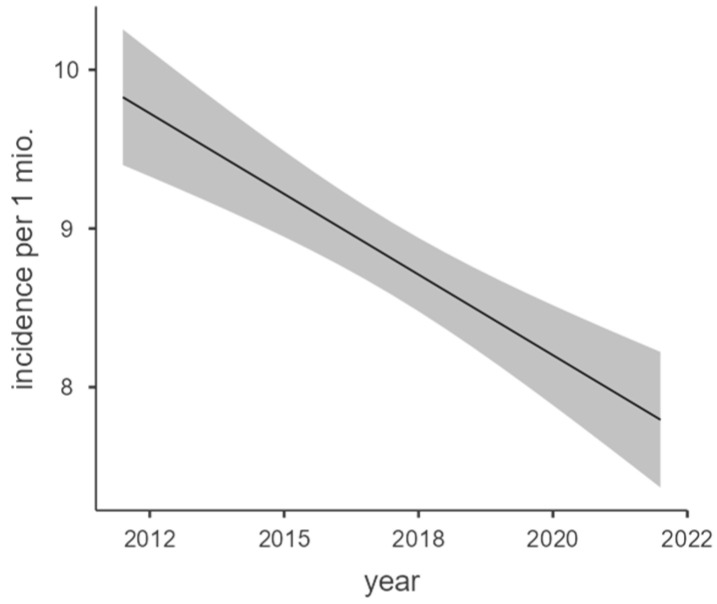
Marginal means plot of overall incidence regression.

The overall incidence development also correlated with the year of report (Figure 2).

The regression model showed a high model fitness with an adjusted R^2^ = 0.798 and significant correlation with F(1,9) = 40.5; *p* < 0.001 and a correlation coefficient of −0.203 per million per year.

**Figure 3:**
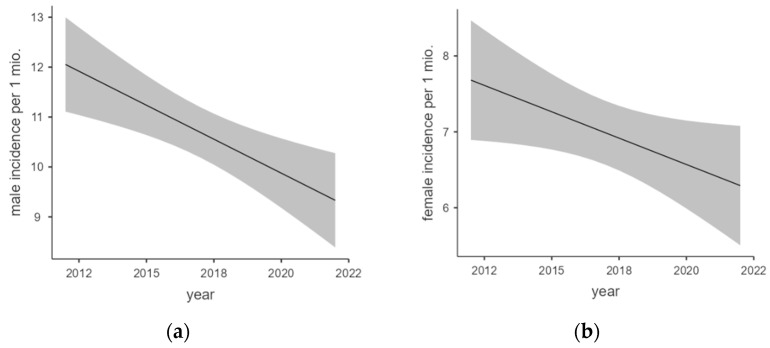
(**a**,**b**) Marginal means plot of male and female incidence regression.

Figure 3a,b show the regression analyses of male and female incidences over the years of report. Both also correlated significantly within the year of report.

Linear regression analysis of male incidence showed a moderate model fitness with an adjusted R^2^ = 0.581. The F-test was calculated as significant with F(1,9) = 14.9; *p* = 0.004 and a regression coefficient of −0.272 per million per year.

Linear regression analysis of female incidence also showed a moderate but slightly lower model fitness with an adjusted R^2^ = 0.383. The F-test was calculated as significant again with F(1,9) = 5.58; *p* = 0.042 and a regression coefficient of −0.139 per million per year.

Overall, a significant trend of incidence decrease can be observed during the study period. This trend is particularly prevalent among the male population, with the female population only showing a slight decrease in overall incidence. An age-dependence can also be observed, with older age groups showing a more heavily decrease in incidence. 

## 4. Discussion

To the best of our knowledge, this is the first study worldwide to provide reliable data on the incidence of kidney infarctions on a nationwide scale in a western country. The knowledge about epidemiology and incidence development provides the basis for further studies and the evaluation of their medical and economic impact. While previous studies focused on patient groups at increased risk, thereby overestimating the incidence, we were able to acquire data including healthy individuals with less a priori risk. Sample bias could therefore be eliminated. Table 3 displays the results of these previous studies. Due to differences in the detection method of kidney infarctions as well as differences in the reference group, the reported incidences of kidney infarctions differ widely. While Kim et al. report incidences as low as 2.68 to 3.06 per 100,000 person-years in the general Korean population, Hoxie et al. report 1.4% of their patients to have suffered from a kidney infarction [17,18]. Clearly, the morbidity is higher in deceased patients in comparison to the general population. The differences in the reported incidence can therefore be explained by differences in the a priori risk of the respective patients. A fair comparison between the patient collective analyzed in this paper can, therefore, only be made to the collective from the study by Kim et al. Although their analysis was conducted in a similar period compared to ours, they report higher incidences as well as an upward trend, while in Germany we see a rather downward trend of kidney infarctions. Also, in Korea kidney infarctions were more common in women, while in Germany, we saw a higher incidence in men.

The temporal differences within the kidney infarction incidence show a downward trend in Germany. This might be attributable to differences in the a priori risk in the general population. Vascular diseases including atherosclerosis and hypertension, atrial fibrillation and prothrombotic states are generally considered the most important risk factors for kidney infarction [21]. While these risk factors have become increasingly prevalent in recent years, mostly attributable to demographic change as well as changes in health behavior, healthcare professionals have also advanced their treatment strategies, including pharmacologic as well as non-pharmacologic interventions. This includes lower treatment goals for the blood pressure and serum cholesterol, as well as the increasingly popular anticoagulatory therapeutics [22,23]. Blood clotting can be significantly altered pharmacologically, which would lower the risk for kidney infarction. Korea, in comparison, has also adapted the novel guidelines, but prescription strategies have not changed much with reference to direct oral anticoagulants [17]. The different trends in kidney infarction incidence could therefore at least partly be explained by these differences in medication strategy.

In our analysis we see a lower risk in female patients compared to male patients. However, this gender gap tended to close in recent years. The incidence in male patients falls faster than in female ones (−0.272 per million per year vs. −0.139 per million per year). Again, this might be attributed to a changing prescription practice with regard to direct oral anticoagulants. Not only are they prescribed more often to men, they also tend to be less effective in women [24,25]. Therefore, the underlying risk factors leading to more thromboembolic events—and thereby also more kidney infarctions—are treated more rigorously, causing improved prevention in men.

The downward trend in the incidence of kidney infarctions stands in contrast to an overall increase in AKI incidence, underscoring distinct risk factors for kidney infarction [26].

### Limitations

The data presented in this paper was gathered and analyzed with the utmost care. However, it should be interpreted with caution. This is mainly due to the analysis being based on claims data. Therefore, its reliability depends on the quality of the primary data. It is possible that kidney infarctions were incorrectly encoded during the observed time period. This would most likely result in an underestimation of the incidence. Additionally, silent kidney infarctions, i.e., kidney infarctions with small to minimal clinical consequences might not be correctly diagnosed and/or hospitalized, which would also result in an underestimation of the incidence. Also, the data used for this analysis unfortunately do not include individualized data or further data including, e.g., patient history, comorbidities or laboratory values. This impedes the conclusions concerning risk factors that can be drawn from this study. Furthermore, it is possible, although from clinical experience unusual, for a primary care hospital to diagnose a kidney infarction but not be able to treat the diagnosed infarction. This could then lead to a referral to a different hospital and therefore a duplication of the diagnosis could have been transmitted to the authorities. This would result in an overestimation of the reported incidence.

The claims data presented here are registered at the time of the discharge of a patient. Therefore, a patient who suffered from a kidney infarction, e.g., at the end of December and who was discharged from the hospital in January next year would be counted towards the next calendar year, resulting in incorrect results. However, since this occurs during every turn of the year, the effects are likely to even out. This study used data from Germany only. Therefore, generalizability might be impaired. Extrapolating the data for countries with different demographic properties and/or a different healthcare system might misestimate the incidence specific to that country.

## 5. Conclusions

We were able to provide a reliable analysis of kidney infarction incidence via a population-based cohort study. A clear decrease in incidence, especially in the elderly, can be observed. We proposed possible mechanisms explaining this development. Further research is needed to elucidate the exact underlying mechanisms. Kidney infarction remains a rare cause for AKI. However, it should not be forgotten as differential diagnosis in typical clinical scenarios.

## Figures and Tables

**Table 1:** Mean incidence per 1 million inhabitants per year.

	Mean	Standard Deviation	Minimum	Maximum
overall	8.81	0.745	7.59	10
female	6.99	0.745	6.24	8.68
male	10.7	1.14	8.73	12.7
age groups				
<20	0.329	0.191	0.126	0.718
20–29	1.04	0.259	0.616	1.5
30–39	4.93	0.62	3.99	5.8
40–49	11.1	1.06	8.94	12.6
50–59	10.7	0.993	9.14	12.7
60–69	12.9	1.49	10.1	15.7
70–79	20.2	2.21	16.9	23.4
>80	21.1	4.67	14.6	31.9

**Table 2:** Comparison between male and female incidence adjusted by age group.

Age Group	*p*	Effect Size d
<20	0.745	0.141
20–29	0.413	−0.356
30–39	<0.001	4.539
40–49	<0.001	6.719
50–59	<0.001	6.356
60–69	<0.001	2.697
70–79	0.02	1.080
>79	0.189	−0.58

**Table 3:** Comparison between our study and previous studies, comparing methods and results.

Study	Country	Methods	Number of Patients	Patient Collective	Incidence
Kim et al., 2023 [17]	South Korea	Claims data	51,849,591	General population	2.68 to 3.06 per 100,000 person-years
Huang et al., 2007 [19]	Taiwan	Computer tomography	481,540	Emergency department	0.004% of patients
Korzets et al., 2002 [20]	Israel	Computer tomography	151,914	Hospitalized patients	0.007% of patients
Hoxie et al., 1940 [18]	USA	Autopsy	14,411	Deceased patients	1.4% of patients
Paris et al., 2006 [8]	France	intraarterial subtraction angiography	18,287	Patients referred to hypertension unit	0.018% per year
Domanovits et al., 1999 [12]	Austria	Computer tomography	248,842	Emergency department	0.007% of patients

## Data Availability

The datasets generated and/or analyzed during the current study are available from the corresponding author on reasonable request.
